# Prognostic Impact of PD-L1 Expression in Malignant Salivary Gland Tumors as Assessed by Established Scoring Criteria: Tumor Proportion Score (TPS), Combined Positivity Score (CPS), and Immune Cell (IC) Infiltrate

**DOI:** 10.3390/cancers12040873

**Published:** 2020-04-03

**Authors:** Hanno M. Witte, Niklas Gebauer, Daniela Lappöhn, Vincent G. Umathum, Armin Riecke, Annette Arndt, Konrad Steinestel

**Affiliations:** 1 Institute of Pathology and Molecular Pathology, Bundeswehrkrankenhaus Ulm, Oberer Eselsberg 40, 89081 Ulm, Germany; daniela.lappoehn@uni-ulm.de (D.L.); vincentumathum@bundeswehr.org (V.G.U.); annettearndt@bundeswehr.org (A.A.); 2 Department of Haematology and Oncology, Bundeswehrkrankenhaus Ulm, Oberer Eselsberg 40, 89081 Ulm, Germany; arminriecke@bundeswehr.org; 3 Department of Haematology and Oncology, University Hospital of Schleswig-Holstein, Campus Lübeck, Ratzeburger Allee 160, 23538 Lübeck, Germany; niklas.gebauer@uksh.de

**Keywords:** salivary gland carcinoma, immuno-oncology, PD-L1, checkpoint inhibition

## Abstract

Background: Malignant neoplasms of the salivary glands are rare, and therapeutic options are limited. Results from recently published studies indicate a possible use for checkpoint inhibition in a subset of patients, but there are no established criteria for programme cell death ligand 1 (PD-L1) scoring in salivary gland carcinomas (SGCs). Methods: In this retrospective study, we present a cohort of 94 SGC patients with full clinical follow-up. We included 41 adenoid cystic carcinomas (AdCC), 21 mucoepidermoid carcinomas (MEC), 16 acinic cell carcinomas (ACC), 12 adenocarcinomas, not otherwise specified (AC, NOS), 2 epithelial-myoepithelial carcinomas (EMC), one salivary duct carcinoma (SDC), and one carcinoma ex pleomorphic adenoma (CA ex PA). Subsequent histopathological analysis was performed with special emphasis on the composition of the immune cell infiltrate (B-/T-lymphocytes). We assessed PD-L1 (SP263) on full slides by established scoring criteria: tumor proportion score (TPS), combined positivity score (CPS) and immune cell (IC) score. Results: We identified significantly elevated CD3+, TP, CP, and IC scores in AC, NOS compared to AdCC, MEC, and ACC. CPS correlated with node-positive disease. Moreover, AC, NOS displayed IC scores of 2 or 3 in the majority (67%) of cases (p = 0.0031), and was associated with poor prognosis regarding progression-free (PFS) (p < 0.0001) and overall survival (OS) (p < 0.0001). CPS correlated with strong nuclear or null p53 staining in AC, NOS but not in other SGCs. Long-lasting partial remission could be achieved in one AC, NOS patient who received Pembrolizumab as third-line therapy. Conclusions: The current study is the first to investigate the use of established scoring criteria for PD-L1 expression in malignant salivary gland tumors. Our findings identify unique characteristics for AC, NOS among the family of SGCs, as it is associated with poor prognosis and might represent a valuable target for immune checkpoint inhibition.

## 1. Introduction

Comprising 0.5% of all malignant tumors and 3% to 6% of head and neck cancers, malignant tumors of the salivary glands are rare [1]. The rarity as well as the histological diversity of these neoplasms contribute to the fact that their biology is incompletely understood, and therapeutic strategies are lacking, especially for patients with advanced stage disease. The routine therapeutic approach for localized salivary gland carcinoma (SGC) is complete surgical excision, if necessary, along with complete or radical neck dissection [2]. If tumors are deemed primarily unresectable, definitive radiotherapy may be used with or without chemotherapy [3,4,5]. Systemic therapy is also the method of choice for patients who are ineligible for upfront surgery or radiotherapy due to comorbidities or as a palliative approach in patients presenting with metastatic disease. However, sparse available data on systemic therapy regimens in salivary gland cancer show, at best, a moderate benefit but considerable toxicity [2,6,7]. Molecular characterization of salivary gland cancers revealed *TP53* mutations as well as therapeutically addressable alterations (such as *ERBB2, PIK3CA, ETV-NTRK3)* in a subset of tumors. However, approved compounds are, so far, only available for a minority of these molecular targets [8,9].

Immune checkpoint inhibition constitutes a well-established approach in the treatment of non-small cell lung cancer (NSCLC) and small cell lung cancer (SCLC), malignant melanoma (MM), urothelial carcinoma (UC), head and neck squamous cell carcinoma (HNSCC), and Hodgkin lymphoma (HL) in a relapsed or refractory setting [10,11]. Since tumor cells evade the cytotoxic T-cell-response by surface expression of modulatory checkpoint proteins, immunohistochemistry (IHC) for PD-L1 expression on tumor and/or immune cells has been proven to be a prognostic biomarker for the response to checkpoint inhibition [12]. However, different scoring methods and cutoffs for the various IHC assays and tumor entities have, so far, been established.

For malignant salivary gland tumors, the first results from the KEYNOTE-028 study indicated a possible therapeutic role for pembrolizumab in a small cohort of 26 patients [13]. In total, 12% of the patients with PD-L1 expression showed a confirmed objective response (three cases of partial remission) with a median duration of four months (range, 4 to 21 months) and a tolerable safety profile [13]. However, the cutoff for PD-L1 expression (clone 22C3) was chosen as ≥1% of a PD-L1-positive tumor or stromal cells based on studies from NSCLC and gastric cancer [14,15]. In a recent study by Vital et al. applying the identical 1% cutoff for PD-L1 positivity (clone SP142), the authors identified 17% of PD-L1 positive SGC (28 of 167 cases) and 20% of tumors with PD-L1-positive infiltrating immune cells (33 of 167 cases) over all histological subgroups [16]. In a smaller cohort of 47 patients, Harada et al. found PD-L1 positivity (clone name not given) in 51.1% of malignant salivary gland tumors using a cutoff of 5% tumor cells with membranous PD-L1 staining [17]. In both latter studies, PD-L1 positivity had a prognostic value, but the predictive relevance of the findings with respect to a possible use of checkpoint inhibitors in these patients remains unclear since different antibodies and cutoffs have been applied. Up to now, there is no study employing the established scoring criteria for PD-L1 expression that are in routine use for other malignancies (TPS, CPS, or IC) in a representative cohort of malignant salivary gland tumors.

With respect to a possible therapeutic use of checkpoint inhibition in SGC, Rodriguez et al. combined pembrolizumab with the histone-deacetylase-inhibitor vorinostat in 25 patients with HNSCC as well as 25 patients with SGC. The combination showed activity in HNSCC with fewer responses in SGC. The group of malignant salivary gland tumors in that study included adenoid cystic carcinoma, acinic cell carcinoma, and mucoepidermoid carcinoma [18].

The aim of the present study was, therefore, (i) to analyze the inflammatory infiltrate in tissue samples from a large and well-characterized cohort of SGC, (ii) to assess TP, CP, and IC scores, and (iii) to correlate these results with the clinic-pathological characteristics of this cohort. The results might identify SGC subgroups with poor clinical outcome after exhaustion of established treatment options and a high probability of response to checkpoint inhibitors.

## 2. Results

### 2.1. Clinicopathologic Characteristics

**Figure 1:**
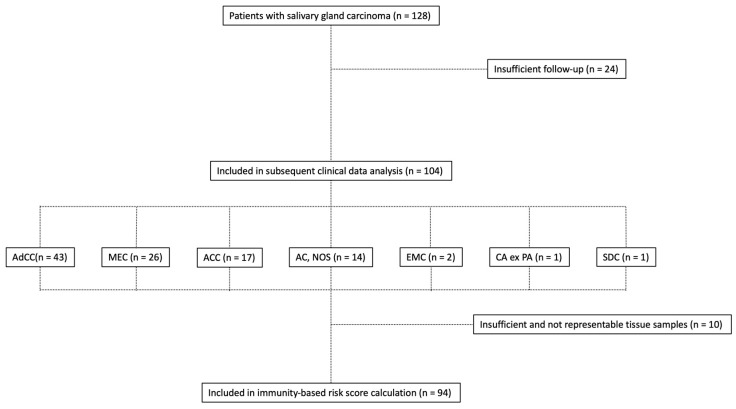
Flowchart depicting the composition of the study group.

**Figure 2:**
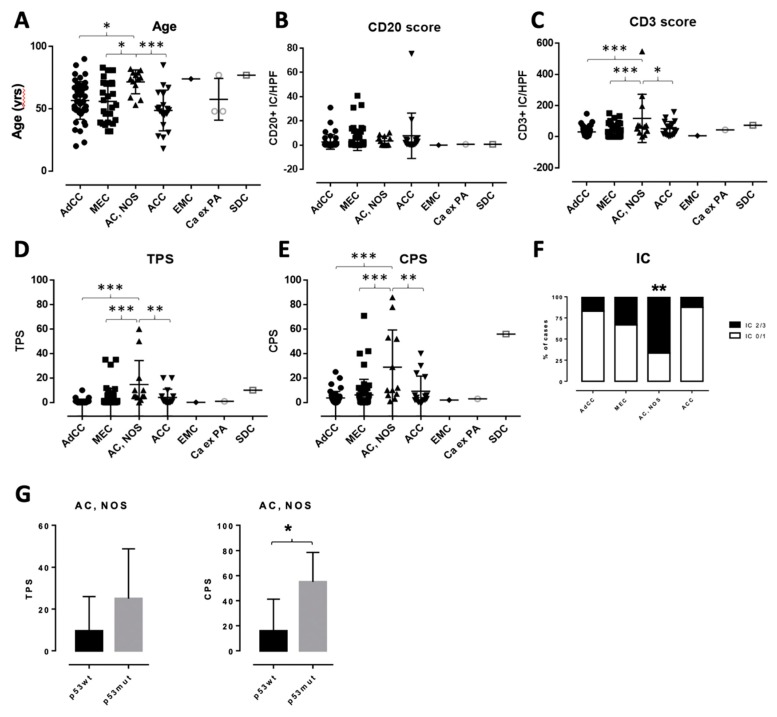
Graphs depicting the results for patient age (**A**), CD20^+^ score (**B**), CD3^+^ score (**C**), TPS (**D**), CPS (**E**), and IC (**F**). (**G**), TPS and CPS values in p53wt and p53mut cases of AC, NOS. *p < 0.05, **p < 0.01, ***p < 0.001. HPF, high power field.

Baseline characteristics of patients with SGC included in the current study are briefly summarized in Table 1. Composition of the study group is depicted in Figure 1. Patients with AC, NOS presented at a significantly higher age (median 74.0 years, range 53–82 years) compared to AdCC (56.0 years, range 20–90 years) and MEC patients (55.0 years, 32–83 years, both *p* < 0.05, Figure 2A) or ACC patients (49.0 years, 18–69 years, *p* < 0.001). A total of 44 patients included in the current study were male (46.8%). The median body mass index (BMI) was 26.2 kg/m^2^ (range: 17.0 to 45.5 kg/m^2^). For 89 of 94 patients (94.6%), the Eastern Cooperative Oncology Group (ECOG) performance Status was 0–2 at the time of diagnosis.

Nearly half of patients (*n* = 40, 42.6%) presented with advanced stage disease (Union for international cancer control (UICC) stages IV A/B/C) at an initial diagnosis. Metastatic disease (UICC stage IV C) could be detected in 17 cases (18.0%). Particularly in patients with AC, NOS, advanced stage disease was more common when compared with the other entities of SGC included in the current study.

In most cases MEC, AC, NOS, and ACC were localized in the parotid gland or other major salivary glands, while AdCC localization was almost evenly distributed between major and minor (palatinal) salivary glands. Lymph-node positive disease was more frequently detected in patients with AC, NOS (nodal status N+, *p* < 0.01).

### 2.2. Histopathological Assessment

**Figure 3:**
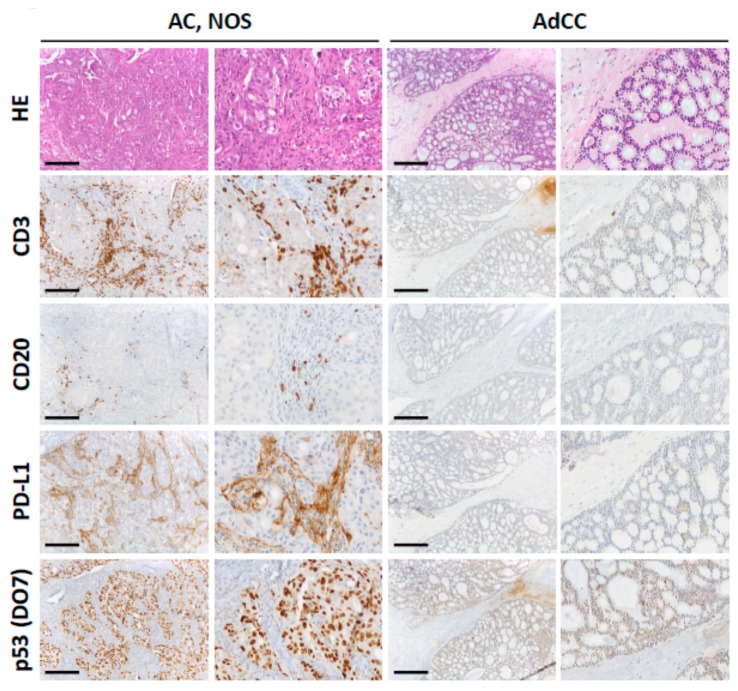
Microphotographs of the immunohistochemistry (IHC) analyses for cluster of differentiation (CD) 3, CD20, programme cell death ligand 1 (PD-L1), and p53 (DO7) in two representative cases of Adenocarcinoma, not otherwise specified (AC, NOS) (case no. 9546/12, left panels including higher magnification) and Adenoid cystic carcinoma (AdCC) (case no. 8738/14, right panels). There is a higher number of tumor-associated lymphocytes (CD3/CD20) and higher PD-L1 expression (Tumor proportional score (TPS) 10, combined positivity score (CPS) 53, immune cell score (IC) 3) in AC, NOS compared to AdCC (TPS 4, CPS 6, IC 1). AC, NOS shows strong nuclear expression of p53 (DO7) while only a few nuclei are positive in AdCC. Scale bar: 200 µm.

We investigated tissue samples of malignant salivary gland tumors from a total of 94 patients (Table 1). Histopathological characteristics of the study group are outlined in Table 2. Diagnostic specimens were obtained from primary tumors when possible: parotid gland (*n* = 41; 43.6%), other salivary glands (*n* = 41; 43.6%). In cases when no primary tumor could be assessed, samples from nodal/distant metastases were evaluated (*n* = 12, 12.8%). Representative immunohistochemical findings for AC (NOS) and AdCC are demonstrated in Figure 3. 

### 2.3. Characterization of the Inflammatory Infiltrate in Salivary Gland Tumors

T- and B-lymphocytic infiltrate was quantified by the use of immunohistochemistry for CD3 and CD20 in three high-power fields (Area: 0.921 mm^2^), respectively. Only very few CD20+ B lymphocytes were detected with/between the tumor cells, and there were no significant differences in the number of tumor-associated CD20^+^ B-lymphocytes between histologic subtypes. There was a statistically significant elevated number of CD3+ T lymphocytes in AC, NOS, when compared to AdCC and MEC (each *p* < 0.001) or ACC (*p* < 0.05), respectively (Figure 2A–C, analysis of variance (ANOVA)). There was also a high number of tumor-associated CD3+ T-lymphocytes in salivary duct carcinoma (SDC). However, the number of cases for this entity was too small for an in-depth statistical evaluation.

### 2.4. Evaluation of PD-L1 Scoring

Only a minority of investigated SGC showed detectable PD-L1 expression on tumor cells (Figure 3). Two cases of AC, NOS were scored with a TPS above 50. However, in AC, NOS, overall TP scores were significantly higher compared to AdCC and MEC (each *p* < 0.001) or ACC (*p* < 0.01), respectively (Figure 2D, ANOVA). There was no significant association between TPS and node-positive disease across all investigated cases (*p* = 0.0792, *t*-test).

One case of MEC and four cases of AC, NOS were scored with a CPS above 50. CPS was significantly higher in AC, NOS compared to AdCC and MEC (each *p* < 0.001) or ACC (*p* < 0.01), respectively (Figure 2E, ANOVA). CPS was significantly associated with node-positive disease (CPS: 8.037 ± 2.274 in node-negative vs. 25.34 ± 7.196 in node-positive disease, *p* = 0.0106, t-test).

There was a significant higher proportion of tumors with IC of 2 or 3 (more than 5% of tumor area) in AC, NOS compared to all other entities (Figure 2F, *p* = 0.0031, Fisher’s exact test). There was no significant association between the IC score and node-positive disease across all investigated cases (*p* = 0.2283, Fisher’s exact test).

### 2.5. P53 (DO-7) Immunohistochemistry

IHC for p53 (DO-7) showed strong nuclear or null p53 staining in four of the five AC, NOS cases with the highest CPS (Figure 3 and Table 2). Only one case of AdCC showed strong nuclear p53 staining. All other SGCs displayed physiologic staining patterns for p53. While there was no significant difference in TPS, the CPS in AC, NOS cases with p53 overexpression or the null staining pattern was significantly higher when compared to the cases with a physiologic p53 staining pattern (Figure 2G, *p* = 0.0277). 

### 2.6. Therapeutic Characteristics and Clinical Outcome

The majority of patients underwent surgical resection after their initial diagnosis (*n* = 85, 90.4%) and 37 patients (39.3%) proceeded with subsequent radiotherapy. Initial radiotherapy was performed in 44 cases (46.8%). Systemic cytoreductive treatment options were implemented in 36 patients. In those cases, systemic platinum-based chemotherapy was the treatment strategy that was applied most frequently (*n* = 34, 57.6% of all systemic therapeutic approaches (*n* = 59) performed in the study). Immunotherapeutic approaches based on PD-1/PD-L1 blockage were conducted in three patients with SGC. Treatment modalities and clinical outcome of the present study cohort are summarized in Table 3. 

After the initial surgical resection, the CR rate was 51.8% (44/85 cases). By means of consecutive radiotherapy, the CR rate could be elevated up to 64.9% (61/85 cases, 17 cases added, data not shown). Sole radiotherapy was able to reach a response rate of 57.1% (4/7 cases). Due to chemotherapy in combination with or without targeted therapeutics such as cetuximab, a CR rate of 6.3% was reached in patients with advanced stage disease (UICC IV A/B/C) while PR was shown in 20 cases (33.9%) and SD was shown in 32.2% of SGC patients (19/59 cases). 

**Figure 4:**
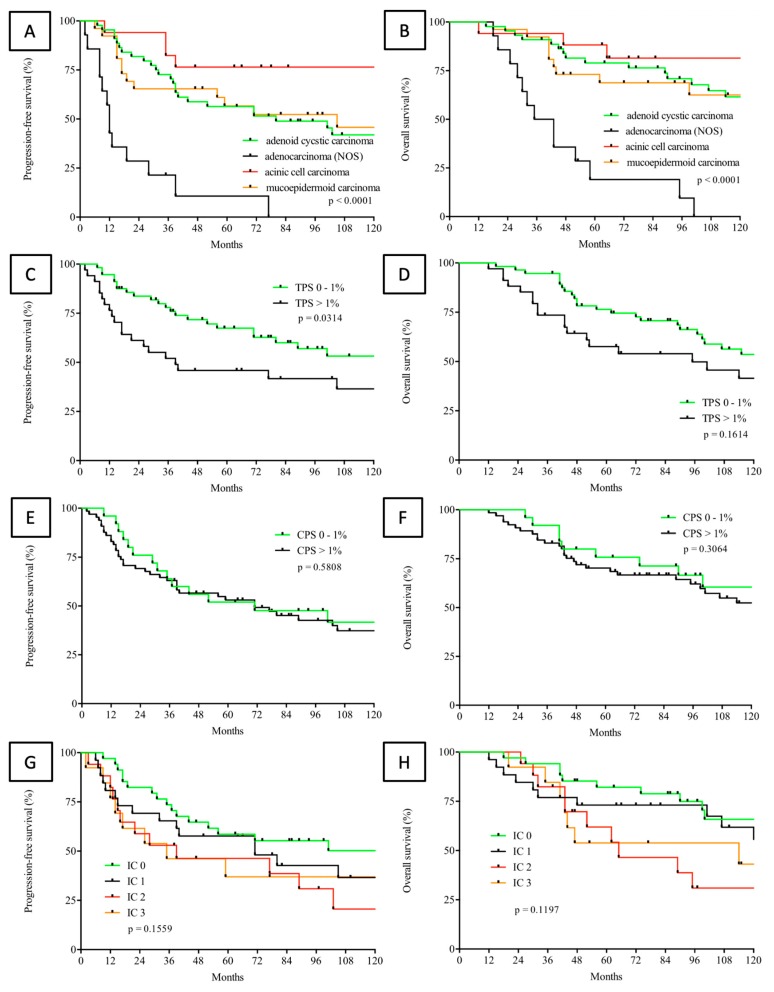
Comparative Kaplan-Meier analysis over all entities of salivary gland carcinomas included in the study regarding progression-free (PFS) (**A**) and overall survival (OS) (**B**). PFS (**C**,**E**,**G**) and OS (**D**,**F**,**H**), according to the immunity-based risk scores TPS (log-rank test, TPS cut-off >1% versus 0–1%, C, D), CPS (log-rank test, CPS cut-off > 1% versus 0–1%, E, F) and IC (log-rank test, IC 0 versus 1 versus 2 versus 3, G, H) in patients with salivary gland carcinoma.

Median follow-up of patients included in the current study was 89.5 months (range, 12 - 240 months). Comparative survival analysis found AC, NOS to be associated with the poorest outcome for PFS (*p* < 0.0001) and OS (*p* < 0.0001) of all SGC in the present study cohort (Figure 4A,B). Kaplan-Meier analysis revealed TPS to be the only possible predictor of PFS (HR = 2.044, 95%CI = 1.066–3.919, *p* = 0.0314), but not OS (HR = 1.611, 95%CI = 0.929–2.792, *p* = 0.1614). Upon further analyses regarding the impact of TPS, CPS, and IC on survival outcome, there were no significant findings (Figure 4C–H). In this case, it must be mentioned that patients with SGC included in the study did not received immunotherapy on a routine basis. Kaplan-Meier survival analysis of the largest cohort represented in the study (AdCC) was stratified according to the UICC stage and TPS, which are depicted in Figure S1.

Immunotherapy was applied in three cases of the study cohort. In two cases of AdCC, nivolumab was administered as the fourth line therapy resulting in partial remissions. Pembrolizumab was given as the third line therapy in a patient suffering from metastatic AC, NOS achieving long lasting partial remission (TTP, 22 months, Table S1).

## 3. Discussion

There are promising results from checkpoint inhibitor therapy in malignant salivary gland tumors, but there is limited data on PD-L1 scoring in these tumors, and no objective scoring criteria has, so far, been evaluated [13,16,17]. While most previous studies focused on the prognostic value of PD-L1 expression, the aim of the present study was to employ established predictive scoring criteria from other tumor entities for the evaluating PD-L1 expression in SGC and to identify entities among SGC that might be promising targets for immuno-oncologic treatment. 

The clinicopathological characteristics of the investigated cohort were comparable to the results from other studies, even though the proportion of AC, NOS was slightly higher when compared to other cohorts [16]. Patients with AC, NOS presented at higher age and a higher frequency of metastatic disease is in line with previous reports [19,20]. Immunohistochemistry on full slides revealed significant higher values for PD-L1 positive tumor cells (TPS) as well as combined positivity score (CPS) in AC, NOS compared to AdCC, MEC, and ACC. This was accompanied by a significant higher number of tumor-infiltrating CD3+ T-lymphocytes in AC, NOS. Given the important role for PD-L1 in developing induced regulatory T cells, it is well conceived that the observed higher PD-L1 expression in AC, NOS, may dampen the antitumoral immune response against tumor cells [21]. This might then contribute to increased tumor aggressiveness, as illustrated by the significant association between node-positive disease and CPS as well as the association between TPS and poor outcome in subsequent Kaplan-Meier survival analysis shown in this study. These results correlate with recent findings in the literature [17,22]. In line with that, a majority of AC, NOS (67%) cases was scored with IC values of 2 or 3, which highlights the tumor area covered by PD-L1 positive immune cells. In the recent study from Vital et al., the authors found a higher frequency of PD-L1^+^ MEC compared to AC, NOS, AdCC, and ACC [16]. However, given the fact that the authors used a different antibody clone (SP142) and adapted the cutoff of 1% PD-L1^+^ tumor cells that had initially been reported for NSCLC and gastric cancer. The comparability between the results is limited [14,15]. Due to the reported heterogeneity of PD-L1 expression in other head and neck malignancies [23], we also see the use of tissue microarray (TMA) slides for the first assessment of PD-L1 expression somewhat critical. Our result of high PD-L1 expression in AC, NOS is in line with data from Mukaigawa et al. who reported PD-L1 expression (1% cutoff, clone E1L3N) in 36% of AC, NOS but only in rare cases of AdCC (2%), MEC (9%), and ACC (0%) [22].

To obtain further information on *TP53* mutation status, additional immunohistochemistry was performed. Four of the five AC, NOS cases with the highest CPS values showed p53 overexpression or null staining pattern, while a similar pattern could only be observed in one single case of AdCC. Accordingly, CPS in AC, NOS with p53 overexpression or null staining pattern was significantly higher compared to cases with physiologic p53 staining pattern. All other tumors showed physiological p53 staining pattern irrespective of PD-L1 expression levels. High frequency of *TP53* gene alterations in AC, NOS, and low frequency in AdCC and MEC has previously been described [24,25]. Mutation or loss of *TP53* in AC, NOS might contribute to the immunogenicity of these neoplasms, which is a mechanism that is thwarted by PD-L1 overexpression and dampening of the host immune response.

In keeping with immunohistochemical findings, subsequent Kaplan-Meier survival analysis was able to identify AC, NOS as the entity to be associated with the poorest clinical outcome (*p* < 0.0001) among the investigated SGC. Established treatment regimens, including surgical resection, radiotherapy, and conventional chemotherapeutic protocols, seem to be less effective in AC, NOS. As already mentioned, the highest expression levels of PD-L1 as well as the most pronounced tumor tissue infiltration of T-lymphocytes were found in AC, NOS. This constellation connotes AC, NOS as the most promising target for immunotherapeutic approaches. Concurrently, survival data for AC, NOS underline the need for improving therapeutic effectiveness compared with other SGC.

Two AdCC and one AC, NOS patient in the present study were treated with pembrolizumab or nivolumab, which results in partial remissions. 

With regard to published data, pembrolizumab treatment was associated with an overall response rate of 12% [13] and 16% [18], respectively. While 10 patients with AC, NOS were treated with pembrolizumab in the KEYNOTE-028 study by Cohen et al., there were no AC, NOS patients included in the study of Rodriguez et al. From this perspective, the effectiveness of pembrolizumab in AC, NOS cases remains unclear. However, the AC, NOS patient from our study achieved a partial response with a duration of 22 months, which exceeds the longest duration of response that had been reported in KEYNOTE-028 [13]. Using the TPS or CPS cutoffs that have previously been established in NSCLC (KEYNOTE-042) or gastric cancer (KEYNOTE-059), this patient might have qualified for frontline pembrolizumab treatment [26,27].

By evaluating the current study cohort, no further statistical inference regarding the efficacy of immunotherapeutic approaches in SGC can be made currently due to a limited sample size of patients receiving pembrolizumab or nivolumab. Moreover, the results from survival analysis have to be interpreted with great caution because of the different therapy regimens that have been applied.

Other possible limitations of this study include its overall limited sample size and retrospective design, which results in the lack of centralized pathology, laboratory, and radiology review for a subset of patients and the potential for fragmentary data and selection bias. It has to be stated that, although diagnoses were carefully re-evaluated, thorough molecular analysis (NGS) of the complete AC, NOS group might have led to re-classification of some tumors as undifferentiated forms of other SGCs. In line with this, the AC, NOS group might be enriched for dedifferentiated or undifferentiated tumors with poor prognosis. However, it might still be useful to perform PD-L1 scoring in such undifferentiated/high-grade tumors because chances are that these patients profit from checkpoint inhibition irrespective of the underlying histologic SGC subtype. Lastly, as another limitation to the study, quantification of B-lymphocytes and T-lymphocytes was performed manually in representative hotspots and not in whole slides/larger areas using software-based methods.

However, our results still suggest that immunotherapeutic approaches hold the potential to play an important role in improving the, as of yet, poor clinical outcome in PD-L1 expressing AC, NOS and other salivary gland cancers. The significant correlation between CPS and nodal disease as well as between TPS and poor PFS supported by findings from other authors emphasizes these results. It would be of great interest to retrospectively apply the scoring criteria used in this case on the patients who received pembrolizumab treatment in the KEYNOTE-028 study or the previously mentioned study of Rodriguez et al. to find out whether the predictive value of PD-L1 assessment would improve [13,18]. Second, it would be of great interest to assess *TP53* mutation status or preferably tumor mutational burden by next generation sequencing (NGS) techniques. 

## 4. Materials and Methods

In this retrospective, single-centre study, we investigated the prognostic value of PD-L1 scoring systems at the initial diagnosis in SGC as a complementary resource for risk stratification. All patients (*n* = 128) with SGC from the Department of Haematology and Oncology as well as the Department of Ear, Nose, and Throat (ENT) of the Bundeswehrkrankenhaus Ulm undergoing surgical resection or cytoreductive treatment between January 2009 and July 2019 were, retrospectively, screened with regard to their inclusion in the current study. Patients with insufficient follow-up (nine patients referred to other centers after primary diagnosis and 15 patients with subsequent loss of follow-up after completion of treatment) or with insufficient or unrepresentative tissue samples (*n* = 10) were excluded (Figure 1). A total of 94 patients undergoing surgical resection and/or cytoreductive therapy could be identified for whom clinical data on prognostic factors and parameters as well as histopathological features had been collected. None of the patients had undergone tumor-specific therapy prior to tissue sampling. Staging was carried out using the 8^th^ edition TNM and the UICC/AJCC staging system for head and neck cancer. 

### 4.1. Patients and Clinicopathologic Data

Clinical information was collected from the original electronic patient files. The collected data included the ECOG (Eastern Cooperative Oncology Group) performance status, staging data, treatment modalities, therapeutic response, pattern of relapse, survival data, and the Charlson Comorbidity Index (CCI). The therapeutic response was evaluated according to ” response evaluation criteria in solid tumors’’ (RECIST) [28,29]. In total, 94 formalin-fixed, paraffin-embedded tissue samples from fully available patients with malignant salivary gland tumors were included in the study. All diagnoses were established and re-evaluated, according to the 4^th^ Edition of the World Health Organization Classification of Head and Neck Tumors [30]. When there was still diagnostic uncertainty, cases were sent out for external reference pathology. Detailed clinicopathological data were retrieved from the respective pathology reports/clinical records and are summarized in Table 1 and Table 2.

### 4.2. Immunohistochemistry

Slides 4 µm in thickness were cut and stained using the following prediluted antibodies from Roche Ventana (Mannheim, Germany): rabbit monoclonal anti-CD3 (2GV6), mouse monoclonal anti-CD20 (L26), mouse monoclonal anti-p53 (DO-7), and rabbit monoclonal anti-PD-L1 (SP263). All antibodies are intended for in vitro diagnostic use (CE-IVD) and were employed following the manufacturer’s protocol on a Ventana BenchMark Ultra immunostainer (Roche, Mannheim, Germany). The sections were deparaffinized in xylene and rehydrated through graded ethanol at room temperature. Incubation with the primary antibodies was performed for 30 minutes at room temperature. After washing, the sections were incubated with biotinylated secondary antibodies. Immunoreactions were visualized using a 3-amino-9-ethylcarbazole as a substrate (Ventana OptiView DAB IHC detection KIT, Ref: 760-700, Mannheim, Germany). Human nonneoplastic tonsillar tissue was used as a positive control for all antibodies.

### 4.3. Quantification of Inflammatory Cells

CD3 positive T- and CD20 positive B-lymphocytes were manually counted in three representative high-power fields (resulting in an area of 0.921 mm^2^) and, from the results, a mean score for each case was calculated. This procedure was performed by two independent pathologists and a consequent mean value was calculated for each case. Consensus assessment was accomplished in cases associated with diverging discrepancy. 

### 4.4. PD-L1 Scoring

PD-L1 scoring (TPS, CPS, and IC) was performed as reviewed by Schildhaus et al. using the following criteria [12]: tumor proportion score, TPS: percentage of viable tumor cells showing partial or complete membrane PD-L1 staining at any intensity (only membranous staining), combined positive/positivity score, CPS: number of PD-L1 staining cells (tumor cells, lymphocytes, macrophages) divided by the total number of viable tumor cells, multiplied by 100, immune cell (IC) score: percentage of tumor area covered by PD-L1^+^ immune cells (4-tiered score: 0: <1%, 1: 1–5%, 2: 5–10%, 3: >10%. Quality and reliability of PD-L1 scoring (TPS, CPS, and IC) has been evaluated through regular interlaboratory ring trials coordinated by the ‘’Qualitätssicherungsinitiative Pathologie’’ (QuIP) GmbH (https://quip.eu) [12].

### 4.5. P53 (DO-7) Scoring

Immunohistochemical staining for p53 (DO-7) was performed in all cases and scored as null (completely negative), strongly positive (+ +, strong nuclear staining signal in all tumor cells), or weakly positive (+, weak to moderate nuclear staining signal in some tumor cells). The null and the strongly positive (+ +) staining patterns were regarded as surrogate markers for null or loss-of-function p53 mutations [31].

### 4.6. Treatment and Assessment

Following baseline staging investigations according to standard procedures, patients over all stages of SGC were treated by surgical resection, radiotherapy, or systemic cytoreductive therapy of the treating physician’s choice with current standard protocols. Treatment response was rated in accordance with established criteria of complete remission (CR) and partial remission (PR). Standard definitions of overall survival (OS) and progression-free survival (PFS) were employed [28]. In addition, the toxicity profile based on National Cancer Institute Common Toxicity Criteria (NCI CTC, version 2.0, Bethesda, MD, USA) was assessed [32,33].

### 4.7. Ethics Statement

All tissue samples were collected for histologic examination and diagnosis purpose and anonymized for the use in this study. Informed consent was, therefore, not needed to be obtained. The local ethics committee of the University of Ulm (reference-no 488-18) approved this. The study was conducted in accordance with the Declaration of Helsinki.

### 4.8. Statistical Methods

All statistical analyses concerning survival data were conducted using GraphPad PRISM 6 (GraphPad Software Inc., San Diego, CA, USA) and SPSS 24 (IBM, Armonk, NY, USA). Progression-free survival (PFS) and overall survival (OS) were calculated from the date of the initial diagnosis. Survival (PFS and OS) was primarily estimated by means of the Kaplan–Meier method and the univariate log-rank test. Differences between continuous variables (Age, TPS, CPS) were analyzed using ANOVA and Tukey’s multiple comparisons test. Differences between categorial variables (N0 vs. N+, IC 0–1 vs. IC 2–3) were analyzed using Fisher’s exact test, respectively. A *p* < 0.05 was regarded as statistically significant.

## 5. Conclusions

Taken together, we show in this paper that application of established scoring criteria for PD-L1 expression (TPS, CPS and IC) identifies AC, NOS as one entity among malignant salivary gland tumors that most likely benefits from immune checkpoint inhibition. Moreover, results of survival analysis exhibit the necessity for innovative treatment options in AC, NOS patients. The predictive value of different (new and established) PD-L1 scoring methods with regard to prognosticate efficacy of immunotherapeutic approaches in salivary gland malignancies should be validated within randomized prospective trials.

## Figures and Tables

**Table 1:** Baseline characteristics for all patients included in the study.

Attribute	Overall Study Group (*n* = 94)	AdCC (*n* = 41)	MEC (*n* = 21)	ACC (*n* = 16)	AC, NOS (*n* = 12)	EMC (*n* = 2)	Ca ex PA* (*n* = 1)	SDC (*n* = 1)
**Male/female**	44 (46.8%)/ 50 (53.2%)	20 (48.8%)/ 21 (51.2%)	9 (42.9%)/ 12 (57.1%)	7 (43.8%)/ 9 (56.2%)	6 (50.0%)/ 6 (50.0%)	1 (50.0%)/ 1 (50.0%)	- 1 (100.0%)	1 (100.0%)/-
**Median age (range), years**	56.5 (18–90)	56.0 (20–90)	55.0 (32–83)	49.0 (18–69)	74.0 (53–82)	46.0 (18–74)	74	77
**BMI (median, range)**	26.2 (17.0–45.5)	26.5 (18.7–34.2)	25.4 (21.3–35.0)	26.1 (22.0–34.9)	24.8 (20.8–45.5)	22.1 (17.0–27.2)	26.3	26.3
**ECOG PS**								
**0 to 2**	89 (94.8%)	39 (95.1%)	19 (90.5%)	16 (100.0%)	11 (91.7%)	2 (100.0%)	1 (100.0%)	1 (100.0%)
**3 + 4**	5 (5.2%)	2 (4.9%)	2 (9.5%)	-	1 (8.3%)	-	-	-
**CCI (median, range)**	4.5 (0–9)	4.0 (0–8)	4.0 (0–8)	2.0 (0–6)	6.5 (4–9)	3.0 (2–4)	4	9
**LDH level**								
**<240 U/L**	62 (65.9%)	28 (68.3%)	12 (57.1%)	12 (75.0%)	6 (50.0%)	2 (100.0%)	1 (100.0%)	1 (100.0%)
**>240 U/L**	32 (34.1%)	13 (31.7%)	9 (42.9%)	4 (25.0%)	6 (50.0%)	-	-	-
B symptoms**								
**Yes**	8 (8.5%)	3 (7.3%)	2 (9.6%)	-	3 (25.0%)	-	-	-
**No**	86 (91.5%)	38 (92.7%)	19 (90.4%)	16 (100.0%)	9 (75.0%)	2 (100.0%)	1 (100.0%)	1 (100.0%)
**UICC/AJCC**								
**I**	20 (21.3%)	9 (22.0%)	5 (23.8%)	5 (31.3%)	-	1 (50.0%)	-	-
**II**	15 (16.0%)	5 (12.2%)	4 (19.0%)	5 (31.3%)	-	1 (50.0%)	-	-
**III**	19 (20.2%)	9 (22.0%)	4 (19.0%)	4 (25.0%)	1 (8.3%)	-	-	1 (100.0%)
**IVA**	15 (16.0%)	8 (19.5%)	3 (14.3%)	1 (6.3%)	2 (16.7%)	-	1 (100.0%)	-
**IVB**	8 (8.5%)	6 (14.6%)	1 (4.8%)	-	-	-	-	-
**IVC**	17 (18.0%)	4 (9.7%)	3 (14.3%)	1 (6.3%)	9 (75.0%)	-	-	-
**Primary localization**								
**GP**	41 (43.6%)	6 (14.6%)	9 (42.9%)	14 (87.5%)	9 (75.0%)	2 (100.0%)	-	1 (100.0%)
**GSM**	15 (16.0%)	9 (22.0%)	4 (19.0%)	1 (6.3%)	1 (8.3%)	-	-	-
**GSL**	4 (4.3%)	2 (4.9%)	2 (9.6%)	-	-	-	-	-
**P**	17 (18.0%)	14 (34.1%)	2 (9.6%)	-	-	-	1 (100.0%)	-
**NC**	5 (5.3%)	4 (9.7%)	-	-	1 (8.3%)	-	-	-
**others**	12 (12.8%)	6 (14.6%)	4 (19.0%)	1 (6.3%)	1 (8.3%)	-	-	-
**Nodal disease**								
**N0**	66 (70.2%)	36 (87.8%)	16 (76.2%)	11 (68.7%)	-	2 (100.0%)	1 (100.0%)	-
**N+**	28 (29.8%)	5 (12.2%)	5 (23.8%)	5 (31.3%)	12 (100.0%)	-	-	1 (100.0%)
**Metastatic disease**								
**M0**	77 (82.0%)	37 (90.3%)	18 (85.7%)	15 (93.7%)	3 (25.0%)	2 (100.0%)	1 (100.0%)	1 (100.0%)
**M+**	17 (18.0%)	4 (9.7%)	3 (14.3%)	1 (6.3%)	9 (75.0%)	-	-	-
**Second malignancy**								
**Yes**	10 (10.6%)	3 (7.3%)	3 (14.3%)	-	4 (33.3%)	-	-	-
**No**	84 (89.4%)	38 (92.7%)	18 (85.7%)	16 (100.0%)	8 (66.7%)	2 (100.0%)	1 (100.0%)	1 (100.0%)

ACC, Acinic cell carcinoma. AC (NOS), Adenocarcinoma. AdCC, Adenoid cystic carcinoma. AJCC, American Joint Committee on Cancer. BMI, Body-Mass-Index. Ca ex PA, Carcinoma ex pleomorphic adenoma. CCI, Charlson Comorbidity Index. ECOG PS, Eastern Cooperative Oncology Group performance status. EMC, Epithelial-myoepithelial carcinoma. GP, Gl. Parotis. GSM, Gl. Submandibularis. GSL, Gl. Sublingualis. LDH, lactate-dehydrogenase. M, metastasis. MEC, Mucoepidermoid carcinoma. N, nodal metastases. NC, nasal cavity and paranasal sinuses. P, palate. SDS, Salivary duct carcinoma. UICC, Union for International Cancer Control. *Myoepithelial carcinoma ex pleomorphic carcinoma; **fever, night sweats, and weight loss.

**Table 2:** Immunohistochemical findings in the study cohort.

Attribute	Overall Study Group (*n* = 94)	AdCC (*n* = 41)	MEC (*n* = 21)	ACC (*n* = 16)	AC, NOS (*n* = 12)	EMC (*n* = 2)	Ca ex PA (*n* = 1)	SDC (*n* = 1)
**Ki-67 (median, range)**	15% (1%–80%)	20% (5%–80%)	5% (2%–15%)	5% (1%–50%)	30% (10%–60%)	7.5%	15%	15%
**p53 (DO7)**								
**null/ + +**	5	1	0	0	4	n.a.	n.a.	n.a.
**+**	89	40	21	16	8	n.a.	n.a	n.a.
**Inflammatory cells** **(median, range)**								
**CD3 ^+^**	31.5 (0–547.7)	22.7 (0–148)	5.3 (0–151)	37.0 (1–158)	66.8 (0.7–547.7)	6	44.3	73.3
**CD20 ^+^**	0.0 (0–75.3)	0.3 (0–31)	0.3 (0–40.7)	1.8 (0–75.3)	1.8 (0–10.3)	0	0.7	0.7
**TPS**								
**mean/median**	4.3/1	0.98/ 1	3.29/ 0.33	4.14/1	14.67/5	0.1/1	1	10
**(range)**	(0–60)	(0–10)	(0–35)	(0–20)	(0–60)			
**TPS < 1**	33 (35.1%)	16	10	6	-	1	-	-
**TPS 1 - 5**	42 (44.7%)	20	8	6	6	1	1	-
**TPS > 5**	19 (20.2%)	5	3	4	6	-	-	1
**CPS**								
**mean/median**	9.8/3.5	3.63/2	6.16/0.5	9.2/3.5	28.92/11	2/2	3	56
**(range)**	(0–86)	(0–25)	(0–71)	(0–40)	(1–86)			
**CPS < 1**	19 (20.2%)	10	6	3	-	-	-	-
**CPS 1 - 10**	52 (55.3%)	25	8	9	7	2	1	-
**CPS > 10**	23 (24.5%)	6	7	4	5	-	-	1
**IC**								
**0–1**	68 (73.1%)	34	14	14	4	1	1	-
**2–3**	25 (26.9%)	7	7	2	8	-	-	1

ACC, Acinic cell carcinoma. AC (NOS), Adenocarcinoma. AdCC, Adenoid cystic carcinoma. Ca ex PA, Carcinoma ex pleomorphic adenoma. CPS, combined positivity score. EMC, Epithelial-myoepithelial carcinoma. IC, immune cells. MEC, Mucoepidermoid carcinoma. **N.a., not applicable.** SDS, Salivary duct carcinoma. TPS, tumor proportion score. (-), none.

**Table 3:** Treatment modalities of patients with salivary gland carcinoma included in the study.

Attribute	Overall Study Group (*n* = 94)	AdCC (*n* = 41)	MEC (*n =* 21)	ACC (*n* = 16)	AC, NOS (*n* = 12)	Others (*n* = 4)
**1^st^ line therapy**						
Surgical resection	85 (90.4%)	35 (85.4%)	19 (90.5%)	16 (100.0%)	11 (91.7%)	4 (100.0%)
Radiotherapy	44 (46.8%)	21 (51.2%)	13 (61.9%)	3 (18.7%)	8 (66.7%)	2 (50.0%)
Chemotherapy (CTX)	11 (11.7%)	3 (7.3%)	3 (14.3%)	2 (12.5%)	4 (33.3%)	-
- CAP	9	3	2	1	3	-
- MFP	-	-	-	-	-	-
- others	2	-	1	-	1	-
**Best response 1^st^ line**						
CR	61 (64.9%)	31 (75.6%)	14 (66.7%)	13 (81.3%)	1 (8.3%)	2 (50.0%)
PR	32 (34.0%)	10 (24.4%)	7 (33.3%)	3 (18.7%)	10 (83.3%)	2 (50.0%)
SD	1 (1.1%)	-	-	-	1 (8.3%)	-
PD	-	-	-	-	-	-
**Lines of therapy**	1.81	1.81	1.62	1.24	2.29	1.5
**(mean, range)**	(1–4)	(1–4)	(1–3)	(1–4)	(1–4)	(1–3)
**Treatment of relapses**						
Surgical resection	22 (23.4%)	12 (29.3%)	5 (23.8%)	-	4 (33.3%)	1 (25.0%)
Radiotherapy	15 (15.9%)	4 (9.7%)	1 (4.8%)	1 (6.3%)	8 (66.7%)	1 (25.0%)
Chemotherapy	34 (36.2%)	18 (43.9%)	5 (23.8%)	3 (18.7%)	7 (58.3%)	1 (25.0%)
- CAP	12	8	1	1	1	1
- MFP	10	5	3	1	1	-
- others	12	5	1	1	5	-
Targeted therapy	14 (14.9%)	7 (17.1%)	2 (9.5%)	1 (6.3%)	4 (33.3%)	-
- mTor inhibition	4	1	1	-	2	-
- EGFR inhibition	3	2	1	-	-2	-
- Immunotherapy	3	2	-	-	1	-
- others	4	2	-	1	1	-
**CTX associated toxicity profile**						
Cytopenia grade III/IV	12 (35.3%)	4 (22.2%)	3 (60.0%)	1 (66.7%)	4 (57.1%)	-
Acute kidney disease	8 (23.5%)	4 (22.2%)	3 (60.0%)	-	1 (14.3%)	-
Sepsis	3 (8.8%)	1 (5.6%)	1 (20.0%)	-	1 (14.3%)	-
Cardiotoxicity	1 (2.9%)	1 (5.6%)	-	-	-	-

ACC, Acinic cell carcinoma. AC (NOS), Adenocarcinoma. AdCC, Adenoid cystic carcinoma. CAP, cisplatin/adriamycin/cyclophosphamide. CTX, chemotherapy. EGFR, epidermal growth factor receptor. MEC, Mucoepidermoid carcinoma. MFP, methotrexat/5-fluorouracil/cisplatin.

## Supplementary Materials

The following are available online at https://www.mdpi.com/2072-6694/12/4/873/s1. Figure S1: Progression-free (A, C) and overall (B, D) survival according to UICC stages (A, B) and immunity-based risk score TPS (log-rank test, TPS cut-off >1% versus 0–1%, C, D) in AdCC patients. Table S1: Patients with SGC undergoing immunotherapeutic approaches included in the study.
