# Supplementary material for: Prognostic Impact of PD-L1 Expression in Malignant Salivary Gland Tumors as Assessed by Established Scoring Criteria: Tumor Proportion Score (TPS), Combined Positivity Score (CPS), and Immune Cell (IC) Infiltrate
Source: Cancers (Basel). 2020 Apr 3;12(4):873. doi: 10.3390/cancers12040873 (PMC7226358; doi:10.3390/cancers12040873)
Supplement: Supplementary file 1 [file cancers-12-00873-s001.pdf]

Supplementary Materials: Prognostic impact of PD-L1 expression in malignant salivary gland tumors as assessed by established scoring criteria: tumor proportion score (TPS), combined positivity score (CPS) and immune cell (IC) infiltrate

Hanno M. Witte, Niklas Gebauer, Daniela Lappöhn, Vincent G. Umathum, Armin Riecke, Annette Arndt, Konrad Steinestel

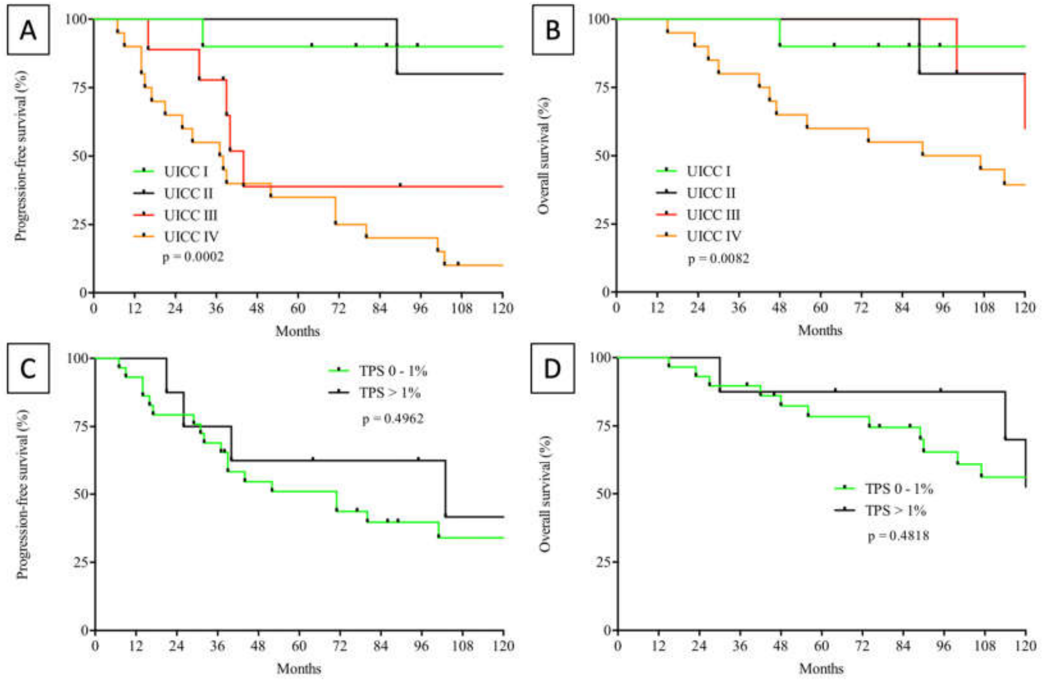

Figure S1. Progression-free (A, C) and overall (B, D) survival according to UICC stages (A, B) and immunity-based risk score TPS (log-rank test; TPS cut-off >1% versus 0 - 1%; C, D) in AdCC patients.

Table S1. Patients with SGC undergoing immunotherapeutic approaches included in the study.

Diagnosis	Sex	Age	Prior lines	Regimen	Best response	TTP (months)	TPS	CPS	IC	Last status
AdCC	m	33	3	Nivolumab	PR	Sus. remission	10%	6	2	Alive remission
AdCC	m	48	3	Nivolumab	PR	20	10%	29	3	Dfd
AC, NOS	m	53	2	Pembrolizumab	PR	22	5%	10	1	Dfd

AC (NOS), Adenocarcinoma; AdCC, Adenoid cystic carcinoma; CPS, combined positivity score; Dfd, died from disease; IC, immune cells; m, male; PR, partial remission; sus. Remission, sustained remission; TPS, tumor proportion score; TTP, time to progress.
